# In Vitro Digested Nut Oils Attenuate the Lipopolysaccharide-Induced Inflammatory Response in Macrophages

**DOI:** 10.3390/nu11030503

**Published:** 2019-02-27

**Authors:** Anke Katharina Müller, Lisa Schmölz, Maria Wallert, Martin Schubert, Wiebke Schlörmann, Michael Glei, Stefan Lorkowski

**Affiliations:** 1 Department of Nutritional Biochemistry and Physiology, Institute of Nutritional Sciences, Friedrich Schiller University Jena, Dornburger Straße 25, 07743 Jena, Germany; anke.katharina.mueller@uni-jena.de (A.K.M.); lisa.schmoelz@uni-jena.de (L.S.); maria.wallert@uni-jena.de (M.W.); m.schubert@uni-jena.de (M.S.); 2 Competence Cluster for Nutrition and Cardiovascular Health (nutriCARD), Halle-Jena-Leipzig, 07743 Jena, Germany; michael.glei@uni-jena.de; 3 Department of Nutritional Toxicology, Institute of Nutritional Sciences, Friedrich Schiller University Jena, Dornburger Straße 24, 07743 Jena, Germany; wiebke.schloermann@uni-jena.de

**Keywords:** Nuts, oleic acid, inflammatory response, fatty acids, macrophages

## Abstract

Nut consumption is known for its health benefits, in particular in inflammatory diseases. A possible mechanism for these effects could be their beneficial fatty acid composition. Nuts mainly contain mono- and polyunsaturated fatty acids, which have anti-inflammatory properties. However, studies investigating the effects of nut extracts on inflammatory processes on the molecular level are rare. We therefore prepared oily nut extracts after in vitro digestion and saponification of the fat-soluble constituents. Besides chromatographic analysis, cell culture experiments were performed using murine macrophages (RAW264.7) to study the capacity of different nut extracts (hazelnut, almond, walnut, macadamia, and pistachio) to modulate inflammatory processes. Oleic acid was the main fatty acid in hazelnut, almond, macadamia, and pistachio extracts. Both oily nut extracts and pure oleic acid significantly reduced the LPS-induced expression of iNos, Cox2, Tnfα, Il1β, and Il6 mRNAs. iNos protein expression was down-regulated followed by reduced nitric oxide formation. Thus, nut extracts at concentrations achievable in the digestive tract inhibit the expression and formation of inflammatory mediators in macrophages. Hence, a beneficial contribution of nut consumption to inflammatory diseases can be assumed. We are convinced that these results provide new insights on the molecular mechanisms involved in the health-beneficial effects of nuts.

## 1. Introduction

Nuts are a substantial part of the Mediterranean diet, and their intake is associated with health-promoting effects [1]. Nuts are energy dense (up to 26.8 kJ/g) mostly due to their high fat content [2]. With their additional high content of dietary fiber, minerals, vitamins, and mono- and polyunsaturated fatty acids, nuts can contribute to a healthy diet [3]. However, the individual nut varieties are very different in their nutrient composition, as shown in, for example, the fat content (50–70%). Besides this, a high fiber content, especially in almonds and hazelnuts [4], is discussed to contribute to the reported inverse association of nut consumption and overweight [5]. Since different types of nuts vary in the composition of their ingredients, guidelines recommend to eat a variety of nuts up to 42.5 g/day [6]. Although the anti-inflammatory effects of nut have been reported, less is known about the underlying molecular mechanisms. Oleic acid (OA), a monounsaturated fatty acid (MUFA), abundant in most of the nuts studied here, inhibits endothelial activation [7] and reduces the pro-inflammatory response induced by lipopolysaccharides (LPS) in murine microglial cells and macrophages [8,9].

Macrophages play a major role in inflammatory diseases by secreting pro-inflammatory mediators and thus maintaining an inflammatory environment [10]. The inflammatory response is an interplay of pro- and anti-inflammatory processes, and resolving mechanisms lead, for example, to a decline in pro-inflammatory mediators like cytokines, including interleukins and tumor necrosis factors (TNF) [11], a controlled release of nitric oxide [12], or an increase in anti-inflammatory mediators such as interleukin (IL) 10 [13]. Nitric oxide is produced by the enzyme nitric oxide synthase (NOS). Besides constitutively expressed isoforms of NOS in endothelial and neuronal tissue, the inducible isoform (iNOS) plays a major role, especially in immune cells. In inflammatory processes, iNOS expression is induced by regulatory proteins, such as cytokines, or microbial stimuli, such as LPS [12]. TNFα is a primary mediator of inflammatory processes, which is involved in immune cell regulation. IL1β acts pro-inflammatory by promoting the expression of mediators, such as the constitutively expressed cyclooxygenase (COX) 2 and IL6 [14].

The aim of our study was to examine the effects of in vitro digested and saponified oily nut extracts (ONE) on inflammatory processes in macrophages. In vitro digestion was performed to imitate human gastrointestinal passage. Hence, we investigated the effects of ONEs on the LPS-induced expression and production of different inflammatory mediators.

## 2. Materials and Methods 

### 2.1. Chemicals

If not indicated otherwise, chemicals were obtained from Carl Roth (Karlsruhe, Germany), Sigma-Aldrich (Seelze, Germany), Thermo Fisher Scientific (Schwerte, Germany), or Merck Millipore (Darmstadt, Germany). All chemicals were used as received from the supplier. 

### 2.2. Source of Nuts

The investigated nut varieties were from South Africa (macadamia); California, USA (pistachios, walnuts, and almonds); and Turkey (hazelnuts). All nuts were mature and harvested in 2012. Nuts were hermetically sealed and stored at 4 °C until use. Fresh ground nuts were used for all experiments.

### 2.3. In Vitro Digestion of Nuts and Extraction of Digestion Products

In the body, nuts or nut ingredients undergo digestion. Therefore, an in vitro simulation of the upper digestive tract (mouth, stomach, and small intestine) was performed to imitate the enzymatical digestion as described [15]. The nut samples obtained after digestion contain the substances, which are available for the body after absorption. Extraction of lipophilic absorption products was performed by vortexing and centrifuging (2057 × *g*, 10 min, 4 °C) using n-hexane/chloroform (5:2, *v*/*v*) for three times. Extracts of each sample were evaporated with nitrogen. The final products were oily nut extracts (ONE), which have been used for all subsequent experiments as indicated in the figures.

### 2.4. Saponification of Oily Extracts

Lipids must be available as free fatty acids for use in in vitro experiments to facilitate cellular uptake and intracellular effects. Chemical saponification was used for dissociation of fatty acids from triglycerides.

#### 2.4.1. Saponification of Nut Oils and Extraction of Oily Nut Extracts 

Saponification was performed according to the method of Degen et al. [16], which was optimized for vegetable oils. In brief, 3 ml of 2 M potassium hydroxide (KOH) was added to 200 mg oily extract and incubated for 30 min at 37 °C. Further, pH 4–5 was obtained using hydrochloric acid before extraction using chloroform and sodium chloride. After centrifugation (3570 × *g*, 5 min, room temperature), the lipophilic phase was collected, and water was removed using a sodium sulfate column. After final evaporation with nitrogen, the free fatty acid containing ONEs were used for further experiments.

#### 2.4.2. Verification of Complete Saponification

Complete dissociation by hydrolysis was verified by thin layer chromatography. An unsaponified vegetable oil sample was used as a negative control and a standard mixture consisting of phospholipids, free cholesterol, free fatty acids, triglycerides, fatty acid methyl esters (FAME), and cholesterol esters served as a positive control for the saponified samples. The loaded thin layer plate was placed in a chamber filled with a solvent mixture of n-hexane/diethyl ether/glacial acetic acid (80:20:2, *v*/*v*/*v*) for 30 min. After spraying 2′,7′-Dichlorfluorescein on the dried plate, UV light exposure (245–266 nm) was used to visualize lipid fractions.

### 2.5. Analytical Procedures: Gas Chromatography (GC)

Fatty acids in ONE samples were methylated with methanolic boron trifluoride solution and heated at 100 °C for 5 min, as described previously [17]. Afterwards, FAME were extracted using n-hexane and sodium sulfate columns. To analyze fatty acids ranging from 4 to 26 carbon atoms via GC (GC-17 V3; Shimadzu, Kyoto, Japan) a fused-silica capillary column DB-225ms (60 m × 0.25 mm, i.d. with 0.25 µm film thickness; J&W Scientific, Agilent, Santa Clara, CA) was used. The injector and detector temperatures were 260 and 270 °C, respectively, using H_2_ as the carrier gas. Fatty acid concentrations were expressed as percentage of the total area of all FAME (% of total FAME). For quantification, GC Lab Solution software version 2.3 (Shimadzu) was used. 

### 2.6. Serum Albumin Coupling of Fatty Acids

Bovine serum albumin (BSA) is a widely used vehicle for free fatty acids in in vitro studies [18]. Sodium salts of OA and saponified nut oil extracts were diluted in a mixture of BSA and Krebs Ringer bicarbonate buffer to a ratio of 4:1 (FA/BSA) and a final fatty acid stock concentration of 6 and 8 mM, respectively. Solutions were directly used for incubation or stored at −20°C in a nitrogen atmosphere.

### 2.7. RAW264.7 Macrophage Culture

Murine RAW264.7 macrophages (ATCC, Manassas, VA, USA) were cultivated as described earlier [19]. Cells (2 × 10^6^ per well) were seeded in 24-well plates, cultured for 24 h in a mixture of 2/3 freshly supplemented high glucose DMEM and 1/3 used culture medium and further treated as indicated in the figures. RAW264.7 macrophages were preincubated with medium, 200 µM BSA-coupled ONE, or 200 µM BSA-coupled OA for 4 h followed by coincubation of 100 ng/ml LPS, 200 µM BSA-coupled ONE, or 200 µM BSA-coupled OA for another 20 h [20]. Control samples were cultured with medium for 4 and further 20 h. Prior to the experiments, treatment with BSA alone was tested and had no effect on the examined parameters. Cells were harvested for further processing as described below.

### 2.8. Cell Viability

Cell viability was determined using MTT assay after 24 h exposure of RAW264.7 cells to various concentrations (0–200 µM) of BSA-coupled ONEs. 3-(4,5-Dimethylthiazol-2-yl)-2,5-diphenyltetrazolium bromide (Amresco, Solon, OH, USA) (0.2 mg/mL) was added to the cell suspension for 4 h, and the formed formazan was dissolved in isopropanol after removal of cell culture medium. Optical density was measured with a FLUOstar Omega microplate reader (BMG Labtech, Ortenberg, Germany) at 570 nm and viability units were normalized to the untreated control.

### 2.9. RNA Isolation and cDNA Synthesis

Qiagen RNeasy Mini kit (QIAGEN, Hilden, Germany) was used to isolate total RNA from samples [21]. cDNA synthesis was performed using Revert Aid First Strand cDNA synthesis kit (Thermo Fisher Scientific) and 500 ng/µL oligo-dT primers as described [22].

### 2.10. Quantitative Real-time PCR (RT-qPCR)

RT-qPCR was run on a LightCycler 480 instrument (Roche Diagnostics, Mannheim, Germany) using Maxima SYBR Green PCR Master Mix (Thermo Fisher Scientific) as described [22]. Primers (Il6, Il1β, Tnfα, iNos, Cox2, peptidylprolyl isomerase B (Ppib)) were purchased from Invitrogen (Karlsruhe, Germany; Supplementary Table S1). Results were analyzed using LightCycler software version 1.5.0.39 (Roche Applied Science, Mannheim, Germany). The fold change of mRNA expression was normalized to the expression of the reference gene Ppib. Quantitative analysis was performed using the 2^-ΔΔCT^ method.

### 2.11. Immunoblotting

Cells were harvested using a non-denaturing buffer and processed for Western blotting as described before [19]. Proteins were separated by SDS-PAGE and transferred to PVDF membrane (Carl Roth). iNos and Cox2 antibodies were diluted with signal enhancer solution (SignalBoost Immunreaction Enhancer kit, Merck KGaA, Darmstadt, Germany), whereas α-tubulin was diluted in hybridization buffer containing 0.5% milk powder and PBS. Primary antibodies mouse anti-iNos (clone 6; 1:2000), rabbit anti-Cox2 (clone EP1978Y; 1:10000), and mouse anti-α-tubulin (clone B-5-1-2; 1:5000) were purchased from BD Biosciences (Heidelberg, Germany), Abcam (Burlingame, CA, USA), and Sigma-Aldrich, respectively. Secondary antibodies (rabbit anti-mouse and swine anti-rabbit both labeled with horseradish peroxidase; 1:5000) were purchased from DAKO (Hamburg, Germany). For detection, Pierce ECL Western Blotting Substrate and CL-XPosureTM Films (Thermo Fisher Scientific) were applied. Blots were analyzed densitometrically using ImageJ software version 1.43u. Relative expression was normalized to α-tubulin.

### 2.12. Quantification of Nitric Oxide Formation Using Griess Assay

Supernatants of RAW264.7-cells were transferred to a 96-well plate, mixed with 130 µL water and 150 µL Griess reagent (Enzo Life Sciences Farmingdale, NY, USA) and incubated in the dark for 30 min at room temperature. Nitrite concentration was measured at 544 nm with a microplate reader. Analyses were performed using MARS data analysis software version 2.41 (BMG Labtech). For calibration, NaNO_2_ concentration series were used.

### 2.13. Statistics

Data are presented either as means ± standard deviation of at least three independent experiments. Statistical differences were analyzed by one-way ANOVA including Tukey post-test using GraphPad Prism® version 7 for Windows (GraphPad Software, San Diego, CA, USA). For all statistical analyses, *p* < 0.05 was considered statistically significant.

## 3. Results

### 3.1. The Five Different ONEs Differ in Their Fatty Acid Composition

The relative quantification of the fatty acid compositions revealed that all analyzed in vitro digested nut oils have an almost identical fatty acid composition compared to the oil extracts before digestion (compare [4]). The MUFA OA (C18:1c9) was the main fatty acid in hazelnut (82.4%), almond (66.4%), macadamia (56.8%), and pistachio extracts (52.9%) (Table 1). Interestingly, macadamia ONE contained a high amount of palmitoleic acid (C-16:1c9, 18.3%). Walnut ONE provided a high content of the *n*-6 polyunsaturated fatty acid (PUFA) linoleic acid (LA) (C-18:2c9,c12, 62.0%), and a considerable content of the *n*-3 PUFA α-linolenic acid (ALA) (αC-18:3c9,c12,c15, 13.4%). Macadamia ONE had the highest amount of saturated fatty acids (SFA), while PUFA was highest in walnut ONE. The *n*-6/*n*-3 PUFA ratio was highest in almond and lowest in walnut ONE. 

### 3.2. ONEs Do Not Affect Viability of RAW264.7 Macrophages

No dose-dependent effects on cell viability were observed for all ONEs in the concentrations used (0–200 µM) (Table 2). 

### 3.3. ONEs and OA Diminish LPS-Induced Expression of iNos in RAW264.7 Macrophages 

**Figure 1:**
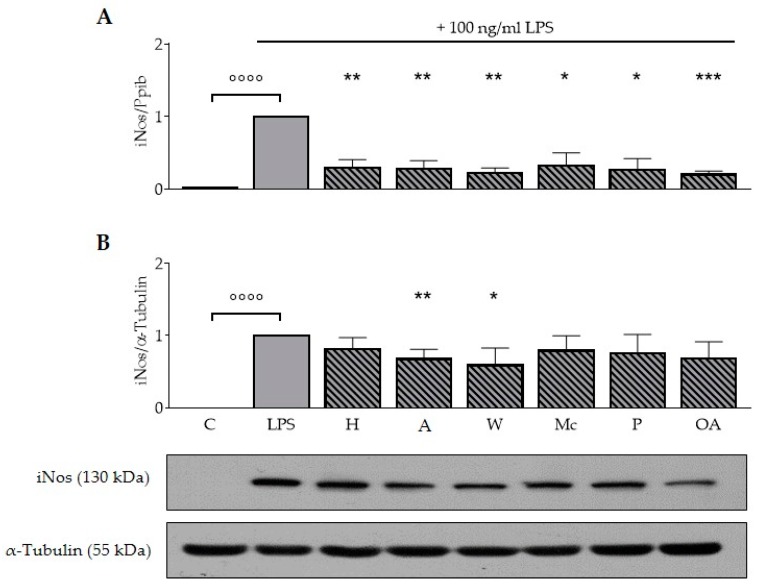
Oily nut extracts and oleic acid diminish lipopolysaccharide-induced expression of iNos mRNA and protein expression in murine RAW264.7 macrophages. Murine RAW264.7 macrophages were preincubated with medium, 200 µM oily nut extracts (ONE), or 200 µM oleic acid (OA) for 4 h followed by a coincubation of 100 ng/ml lipopolysaccharide (LPS) with medium, 200 µM ONEs, or 200 µM OA for additional 20 h. Untreated control samples were cultured with medium for 4 h plus 20 h; values of positive controls (LPS) were defined as 1. Expression of iNos mRNA (RT-qPCR) was normalized to Ppib mRNA expression, whereas iNos protein level (Western blot) was normalized to α-tubulin protein expression. (**A**) ONEs diminished LPS-induced iNos mRNA expression similar to OA. (B) Protein expression was also significantly reduced by the ONEs of almonds and walnuts. Western blots shown here are representative examples of the blots used for densitometry. Error bars display calculated mean expression levels of four independent biological replicates with one (A) or with two technical replicates (**B**). Abbreviations: A, almond; C, control; H, hazelnut; M, macadamia; OA, oleic acid; P, pistachio; W, walnut. Significant differences compared to the untreated control (°°°° *p* < 0.0001) and to the LPS control (* *p* < 0.05, ** *p* < 0.01, *** *p* < 0.001) were obtained by one-way ANOVA.

To determine whether ONEs or OA modulate the immune response in macrophages, we assessed the expression of iNos in RAW264.7 using both RT-qPCR and immunoblotting. In all experiments, the incubation with 100 ng/ml LPS for 20 h resulted in a significant increase of iNos mRNA expression compared to the untreated control (*p* < 0.0001; Figure 1A). All nut extracts and OA reduced the mRNA expression of the inflammatory iNos (*p* < 0.05; *p* < 0.01; *p* < 0.001). ONE of hazelnut, almond, walnut, macadamia, pistachio, and OA reduced iNos levels by 73%, 73%, 79%, 71%, 76%, and 81%, respectively. Expression of the reference gene Ppib did not change during LPS or ONE treatment (data not shown).

In all experiments, the incubation with LPS significantly increased expression of iNos protein (*p* < 0.0001, Figure 1B). Neither all nut samples nor pure OA reduced protein expression levels of iNos significantly. Only ONE of almonds (*p* < 0.01) and walnuts (*p* < 0.05) led to a significant reduction by 33% and 41%.

### 3.4. ONEs and OA Reduce LPS-Induced mRNA Expression of Cox2 in RAW264.7 Macrophages

**Figure 2:**
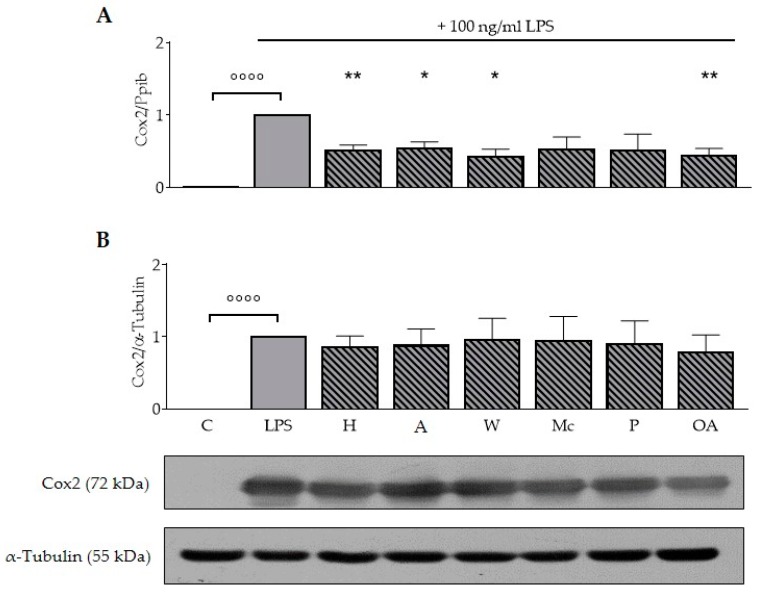
Oily nut extracts and oleic acid decrease lipopolysaccharide-induced expression of Cox2 expression in murine RAW264.7 macrophages. RAW264.7 macrophages were incubated as described in Figure 1, and respective analysis and normalizations have been performed as mentioned before. (A) Oily nut extracts (ONE) of hazelnuts, almonds, walnuts, and oleic acid (OA) significantly reduced lipopolysaccharide (LPS)-induced expression of Cox2 mRNA. (B) Protein expression of Cox2 was not significantly reduced. The Western blots are representative examples of the blots used for densitometry. Error bars display calculated mean expression levels of four independent biological replicates with one (**A**) or with two technical replicates (**B**). Abbreviations: A, almond; C, control; H, hazelnut; M, macadamia; OA, oleic acid; P, pistachio; W, walnut. Significant differences compared to the untreated control (°°°° *p* < 0.0001) and to the LPS control (* *p* < 0.05, ** *p* < 0.01) were obtained by one-way ANOVA.

The data shown in Figure 2A demonstrate that LPS significantly induced expression of Cox2 mRNA (*p* < 0.0001) compared to the untreated control and the LPS-induced mRNA expression levels of Cox2 were reduced after treatment with ONEs of hazelnut (50%), almond (47%), walnut (59%), and OA (57%), respectively (*p* < 0.05; *p* < 0.01). 

Analog to mRNA expression, LPS significantly increased expression of Cox2 protein in all experiments (*p* < 0.0001; Figure 2B). Interestingly, neither ONEs nor OA were able to decrease the LPS-induced protein expression of Cox2 significantly.

### 3.5. ONEs and OA Diminish Cytokine Expression in RAW264.7 Macrophages

**Figure 3:**
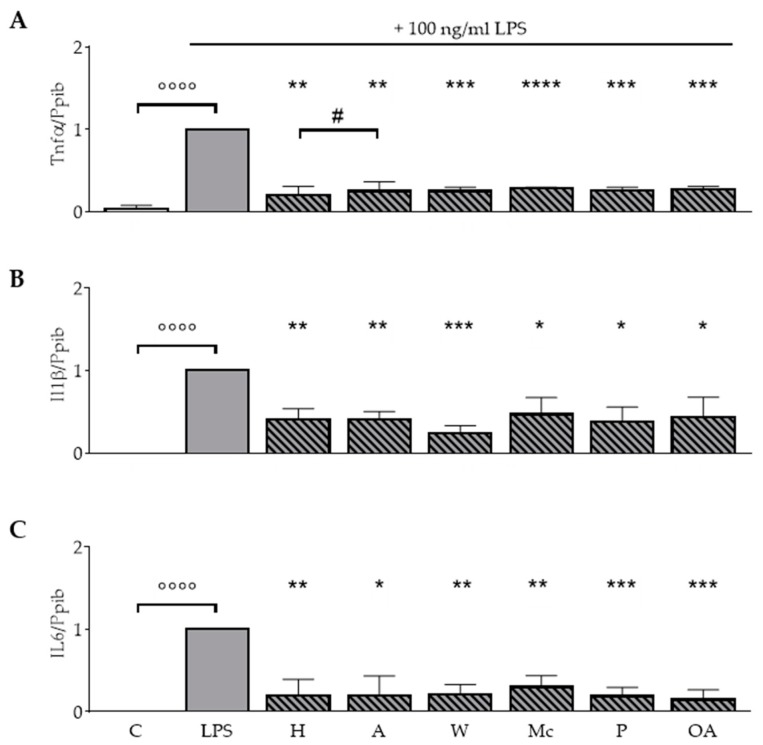
Oily nut extracts (ONE) and oleic acid (OA) strongly block LPS-induced Il1β, Il6, and Tnfα mRNA expression in RAW264.7 macrophages. RAW264.7 macrophages were incubated as described in Figure 1, and respective analysis and normalizations have been performed as mentioned before. (**A**) ONEs and OA significantly reduced LPS-induced expression of Tnfα. (**B**) Expression of Il1β was significantly reduced. (**C**) Expression of Il6 was significantly reduced. Error bars display calculated mean expression levels of 4 (A) to 5 (B,C) biological replicates. Abbreviations: A, almond; C, control; H, hazelnut; M, macadamia; OA, oleic acid; P, pistachio; W, walnut. Significant differences compared to the untreated control (°°°° *p* < 0.0001) and to the LPS control (* *p* < 0.05, ** *p* < 0.01, *** *p* < 0.001, **** *p* < 0.0001) were obtained by one-way ANOVA. # *p* < 0.05, represents significant differences between oily nut extracts.

To investigate the impact of the ONEs on cytokine production during the inflammatory response, LPS-treated RAW264.7 cells were incubated with ONEs of the different nuts or OA. Basal expression levels of Il1β, Il6, and Tnfα mRNA were not influenced by either ONEs or OA (data not shown). Incubation with LPS led to a significant increase of Il1β, Il6, and Tnfα mRNA expression compared to the untreated control (*p* < 0.0001; Figure 3). LPS-induced Tnfα mRNA expression was significantly reduced by all ONEs and OA (*p* < 0.01; *p* < 0.001 *p* < 0.0001; Figure 3A). Levels declined to 18%, 23%, 24%, 29%, 25%, and 27% for the ONEs of hazelnuts, almonds, walnuts, macadamias, pistachios, and OA, respectively. ONE of hazelnuts had significantly stronger effects compared to ONE of almonds. 

The ONEs diminished LPS-induced mRNA expression of the inflammatory cytokine Il1β by 61% (hazelnuts), 61% (almonds), 78% (walnuts), 57% (macadamias), 64% (pistachios), and 61% (OA), respectively (*p* < 0.05; *p* < 0.01; *p* < 0.001; Figure 3B). 

In addition, ONEs and OA significantly blocked the LPS effect on Il6 mRNA expression (*p* < 0.05; *p* < 0.01; *p* < 0.001; Figure 3C). LPS-induced mRNA expression levels were reduced to 15%, 13%, 16%, 27%, 17%, and 12% for the ONEs of hazelnuts, almonds, walnuts, macadamias, pistachios, and OA, respectively. 

### 3.6. ONEs and OA Inhibit LPS-Induced Nitric Oxide Production in RAW264.7 Macrophages

**Figure 4:**
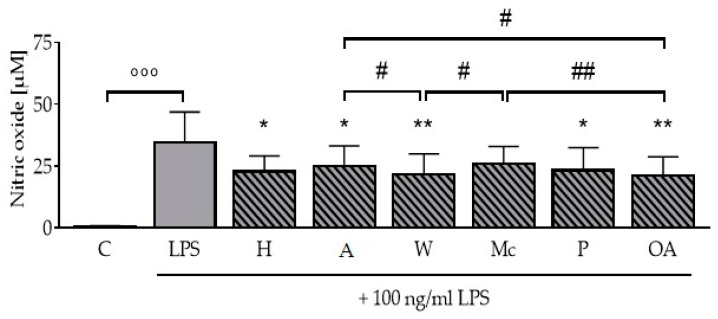
Oily nut extracts (ONE) and oleic acid (OA) decrease formation of nitric oxide in murine RAW264.7 macrophages. RAW264.7 macrophages were incubated as outlined in Figure 1 to perform Griess assays. The incubation with lipopolysaccharides (LPS) led to a significant increase in the production of nitric oxide to 34.7 µM. ONEs significantly reduced the LPS-induced production of nitric oxide. Error bars display calculated means of five independent biologically replicates performed as two technical replicates. Abbreviations: A, almond; C, control; H, hazelnut; M, macadamia; OA, oleic acid; P, pistachio; W, walnut; Significant differences compared to the untreated control (°°° *p* < 0.001) and to the LPS control (* *p* < 0.05, ** *p* < 0.01) were obtained by one-way ANOVA. # *p* < 0.05 and ## *p* < 0.01 represents significant differences between oily nut extracts.

The ONEs of nuts and OA alone had no effect on nitric oxide formation (data not shown). While LPS significantly induced the formation of nitric oxide to 34.7 ± 12.2 µM (*p* <0.001) as assessed by Griess assay compared to the untreated control (Figure 4), the ONEs of hazelnuts, almonds, walnuts, pistachios, and OA significantly reduced the LPS-induced formation of nitric oxide to 22.7 ± 6.4 µM, 24.9 ± 8.1 µM, 21.6 ± 8.3 µM, 23.4 ± 9.1 µM, and 21.0 ± 7.7 µM, respectively (*p* < 0.05; *p* < 0.01). The ONEs of walnuts and OA had both significantly stronger effects compared to the ONEs of almonds and macadamias (*p* < 0.05; *p* < 0.01).

## 4. Discussion

In the work presented here, different parameters were examined, which may shed light on how nuts mediate anti-inflammatory effects. To study this, the impact of nut extracts on inflammatory mediators were measured on mRNA, protein, and functional level in LPS-activated murine RAW264.7 macrophages. Oleic acid was used as comparison, because it is the main fatty acid in most of the ONEs and has previously shown to have anti-inflammatory effects in different cell lines [7,8,9,23].

Bacterial toxins, such as LPS, and cytokines induce iNos and catalyze the formation of nitric oxide from L-arginine in macrophages and microglial cells. Hence, iNos plays a key role in the inflammatory response [24]. In our study, the stimulation of murine RAW264.7 macrophages with LPS led to a significant induction of iNos mRNA and protein expression as expected [19]. All ONEs and OA significantly diminished the LPS-induced expression of iNos on mRNA and partly also on protein level, with no significant difference between the ONEs of different nuts (Figure 1). Almost nothing is known about the effects of the lipophilic fraction of nuts in cellular systems. However, the impact of the main ingredients, namely fatty acids, on molecular processes have been widely studied. Oh et al. (2009) observed a dose-dependent inhibitory effect of OA on LPS-induced expression of iNos mRNA and protein in BV2 microglial cells and confirmed their results in primary rat microglial cells [8]. In RAW264.7 macrophages a treatment with 32 µM OA or LA coupled to BSA reduced iNos protein expression compared to control [9]. In contrast, de Lima and colleagues reported an increase of iNos protein expression after 12 h of incubation with 5 µM OA in macrophages followed by a decrease to control level after 24 h [25].

Besides alterations on iNos mRNA and protein level, we measured the release of nitric oxide. Its production was reduced by all ONEs and conditions tested, except the ONE of macadamia (Figure 4). Interestingly, this reduction in the formation of the inflammatory mediator nitric oxide was stronger on the functional level, while the effects on the iNos protein expression level were less consistent between the ONEs. OA and ONE of walnuts were the most effective treatments tested in this study with significant stronger effects compared to almond and macadamia ONEs. Earlier studies on the formation of nitric oxide showed controversial results. An incubation with 32 µM OA was not able to decrease the amount of nitric oxide in RAW264.7 cells significantly, as reported earlier [9], while de Lima et al. (2006) reported elevated nitric oxide production in murine macrophages after treatment with concentrations from 1 to 100 µM OA, while 200 µM led to a reduction [25]. They explained this decrease by cytotoxic effects, but they used another cell line and application. Other groups working with different cell lines tested higher concentrations up to 600 µM OA with no toxic impact [26,27]. Along with this, no cytotoxic effects have been observed after incubation of RAW264.7 macrophages with 200 µM ONEs in the presented study (Table 2).

Expression of Cox2 is induced in response to inflammatory stimuli [28]. Treatment of RAW264.7 macrophages with ONEs of hazelnut, almonds, walnuts, and OA significantly blocked the LPS-induction of this enzyme on mRNA level (Figure 2). The inhibitory effect of OA on Cox2 mRNA and protein expression was observed in both primary rat microglial cells and murine BV2 microglial cells [8]. According to Chang et al. (2012), 32 µM OA decreased Cox2 protein expression in trend, while prostaglandin E_2_, the Cox2-derived product, was significantly reduced [9]. Another study screening for inhibitors indicated that OA was not able to inactivate Cox2-catalyzed biosynthesis of prostaglandins up to 500 µM [29]. Interestingly, in our study neither ONEs nor OA reduced the LPS-induced expression of Cox2 protein. The stronger effects on mRNA expression could be due to the regulation on the post-transcriptional level [30]. The impact of ONEs and OA on the formation of prostaglandins can be a further target.

Next, we reported a reducing effect of ONEs and OA on the LPS-induced response of the cytokines Tnfα, Il6, and Il1β (Figure 3). Studies in macrophages of different origin also found anti-inflammatory effects of unsaturated fatty acids on the expression and secretion of cytokines such as Tnfα, Il6, and Il1β [9,31,32,33,34].

Grace et al. studied the anti-inflammatory effects and antioxidant activities of lipophilic and hydrophilic extracts from skin and kernels of roasted pistachios in different cell lines [35]. Lipid accumulation and formation of reactive oxygen species were reduced after treatment with both lipophilic and hydrophilic fractions. The anti-inflammatory effect measured via analyses of gene expression of Cox2, iNos, and Il6 was higher for the hydrophilic fractions, possibly due to the presence of polyphenols. As previously reported, polyphenols in nuts and other health-related foods, such as olive oil, mediate anti-inflammatory effects [36]. However, during our work-up procedure (see Section 2.4), polyphenols were eliminated in the ONEs. Therefore, we hypothesize that the fatty acids in the lipophilic fraction of the ONEs contribute to the anti-inflammatory effects. The MUFA OA is an abundant fatty acid in human plasma with physiological concentrations up to 250 µM, depending on the food intake and fasting state [37,38,39]. Hence, we decided to use 200 µM of ONEs and OA to imitate postprandial physiological conditions in our examination of effects mediated by the lipophilic saponified fraction of nuts.

## 5. Conclusions

In conclusion, the results obtained in the present study provide first evidence that oily extracts of different in vitro digested nuts have an anti-inflammatory potential by diminishing the pro-inflammatory response, including iNos and cytokines. In this way, nuts can likely contribute to a healthy diet.

## Figures and Tables

**Table 1:** Fatty acid composition (% of total FAME ^1^) of the oily nut extracts after in vitro digestion.

Fatty Acids	Hazelnut	Almond	Walnut	Macadamia	Pistachio
C-16:0	5.2	6.0	6.4	8.8	11.0
C-16:1c9	0.2	0.4	0.1	18.3	1.0
C-18:0	2.3	1.2	2.7	2.8	1.1
C-18:1c9 (n-9) (OA ^2^)	82.4	66.4	13.9	56.8	52.9
C-18:1c11	1.0	1.3	0.7	3.8	2.3
C-18:2c9,c12 (*n*-6) (LA ^3^)	8.2	23.8	62.0	2.5	30.1
αC-18:3c9,c12,c15 (*n*-3) (ALA ^4^)	0.1	<0.1	13.4	0.1	0.5
C-20:0	0.1	0.1	0.1	2.2	0.1
C-20:1c11 (*n*-9)	0.1	0.1	0.2	2.2	0.3
Σ SFA ^5^	7.8	7.5	9.4	15.4	12.4
Σ MUFA ^6^	83.8	68.2	15.0	81.4	56.7
Σ PUFA ^7^	8.4	24.0	75.5	2.7	30.7
Σ *n*-3 PUFA	0.1	<0.1	13.4	0.1	0.5
Σ *n*-6 PUFA	8.2	23.8	62.0	2.5	30.1
*n*-6/*n*-3 PUFA ratio	90.8	713.6	4.6	18.4	61.5

^1^ FAME, fatty acid methyl esters; ^2^ OA, oleic acid; ^3^ LA, linoleic acid; ^4^ ALA, α-linolenic acid; ^5^ SFA, saturated fatty acid; ^6^ MUFA, monounsaturated fatty acid; ^7^ PUFA, polyunsaturated fatty acid.

**Table 2:** Viable cell number (% of untreated control) of RAW264.7-macrophages after 24 h of incubation with 0–200 µM oily nut extracts of different nut types. Values of untreated controls were set to 100% and data were expressed as means ± SD of three independent experiments.

ONE (µM)	Hazelnut	Almond	Walnut	Macadamia	Pistachio
0	100.0	100.0	100.0	100.0	100.0
1	99.8 ± 13.2	110.9 ± 2.4	93.3 ± 1.9	99.9 ± 3.3	90.8 ± 7.5
5	96.2 ± 6.5	100.5 ± 1.3	96.8 ± 2.4	96.2 ± 4.7	93.8 ± 6.6
10	97.8 ± 11.4	102.3 ± 4.6	97.4 ± 4.1	95.1 ± 5.9	99.3 ± 6.5
50	107.3 ± 12.9	109.2 ± 4.1	98.1 ± 2.6	96.0 ± 1.1	95.4 ± 9.8
75	116.5 ± 35.6	105.6 ± 2.6	100.1 ± 0.6	95.6 ± 7.1	89.6 ± 6.4
100	112.7 ± 15.2	104.0 ± 7.3	102.5 ± 2.6	94.6 ± 6.7	95.7 ± 3.7
200	119.8 ± 28.2	112.4 ± 7.7	93.3 ± 3.5	96.8 ± 6.0	97.4 ± 7.3

## Supplementary Materials

The following are available online at https://www.mdpi.com/2072-6643/11/3/503/s1, Table S1: Primers used for RTq-PCR analyses in the present study. The primers of a pair are located in different exons.
