# Supplementary material for: In Vitro Digested Nut Oils Attenuate the Lipopolysaccharide-Induced Inflammatory Response in Macrophages
Source: Nutrients. 2019 Feb 27;11(3):503. doi: 10.3390/nu11030503 (PMC6471109; doi:10.3390/nu11030503)
Supplement: Supplementary file 1 [file nutrients-11-00503-s001.pdf]

nutrients

Article

Supplementary Material

Supplementary Table S1: Primers used for RTq-PCR analyses in the present study. The primers of a pair are located in different exons.

mRNA	Name	Forward primer/<br>Reverse primer [5′ → 3′]
**Cox2**	Cyclooxygenase 2	TCCCTGAAGCCGTACACATCA/<br>TGGACGAGGTTTTTCCACCA
**Il1β**	Interleukin 1β	TGAAGTTGACGGACCCCAAA/<br>CAGCCACAATGAGTGATACTGCC
**Il6**	Interleukin 6	TCAATTCCAGAAACCGCTATGAA/<br>GGAAGGCCGTGGTTGTCAC
**iNos**	Inducible nitric oxide synthase	GAGCGAGGAGCAGGTGGAA/<br>CCATAGGAAAAGACTGCACCGA
**Ppib**	Peptidylprolyl isomerase B	AAACAGCAAGTTCCATCGTGTCAT/<br>GAAGCGCTCACCATAGATGCTCT
**Tnfα**	Tumor necrosis factor α	AGAAACACAAGATGCTGGGACAGT/<br>CCTTTGCAGAACTCAGGAATGG
